# Compressive streak microscopy for fast sampling of fluorescent reporters of neural activity

**DOI:** 10.1117/1.NPh.12.2.025013

**Published:** 2025-05-22

**Authors:** Changjia Cai, Owen Traubert, Jovan Tormes-Vaquerano, M. Hossein Eybposh, Srinivas C. Turaga, Jose Rodriguez-Romaguera, Eva A. Naumann, Nicolas C. Pégard

**Affiliations:** a University of North Carolina at Chapel Hill, Department of Applied Physical Sciences, Chapel Hill, North Carolina, United States; b University of North Carolina at Chapel Hill, Joint Department of Biomedical Engineering, Chapel Hill, North Carolina, United States; c Duke University, Department of Biomedical Engineering, Durham, North Carolina, United States; d Duke University, Department of Neurobiology, Durham, North Carolina, United States; e HHMI Janelia Research Campus, Ashburn, Virginia, United States; f University of North Carolina at Chapel Hill, Department of Psychiatry, Chapel Hill, North Carolina, United States; g University of North Carolina at Chapel Hill, Department of Cell Biology and Physiology, Chapel Hill, North Carolina, United States; h University of North Carolina at Chapel Hill, Neuroscience Center, Chapel Hill, North Carolina, United States; i University of North Carolina at Chapel Hill, Carolina Institute of Developmental Disabilities, Chapel Hill, North Carolina, United States; j University of North Carolina at Chapel Hill, Carolina Stress Initiative, Chapel Hill, North Carolina, United States

**Keywords:** calcium imaging, voltage imaging, streak imaging, compressive microscopy, computational imaging

## Abstract

**Significance:**

*In vivo* one-photon fluorescence imaging of calcium and voltage indicators expressed in neurons enables noninvasive recordings of neural activity with submillisecond precision. However, data acquisition speed is limited by the frame rate of cameras.

**Aim:**

We developed a compressive streak fluorescence microscope to record fluorescence in individual neurons at high speeds (≥200 frames per second) exceeding the nominal frame rate of the camera by trading off spatial pixels for temporal resolution.

**Approach:**

Our microscope leverages a digital micromirror device for targeted illumination, a galvo mirror for temporal scanning, and a ridge regression algorithm for fast computational reconstruction of fluorescence traces with high temporal resolution.

**Results:**

In simulations, the ridge regression algorithm reconstructs traces of high temporal resolution with limited signal loss. Validation experiments with fluorescent beads and experiments in larval zebrafish demonstrate accurate reconstruction with a data compression ratio of 10 and accurate recordings of neural activity with 200- to 400-Hz sampling speeds.

**Conclusions:**

Our compressive microscopy enables new experimental capabilities to monitor activity at a sampling speed that outpaces the nominal frame rate of the camera.

## 1 Introduction

Neurons fire action potentials across the nervous system that drive animal behavior. A preferred approach to capture neural activity in living animals, with single-cell resolution and across many neurons in parallel, relies on imaging fluorescent reporters of neural activity using fluorescence microscopy.^1^^–^^3^ Utilizing the nonlinear effect of two-photon excitation, the current state-of-the-art, two-photon microscopy,^4^^,^^5^ limits the out-of-focus fluorescence artifacts and yields clear images through hundreds of micrometers of brain tissue. However, the acquisition speed of two-photon microscopes is limited by design to sampling fluorescence inside a diffraction-limited focused spot that must be mechanically scanned across the entire field of view (FOV) to generate an image. Recently, several groups have explored scaling up two-photon microscopy’s speed with different imaging techniques, including sampling multiple locations simultaneously,^6^ splitting laser pulses temporally,^7^ and exciting fluorescence along lines.^8^

Alternate imaging solutions based on one-photon fluorescence microscopy are simpler to implement, more cost-effective, and easier to scale than^2^ two-photon techniques. However, one-photon fluorescence microscopy is subject to out-of-focus fluorescence artifacts that introduce noise and lower contrast, to optical aberrations in brain tissue that degrade spatial resolution, and to optical scattering that substantially reduces accessible imaging depths.

Perhaps the most widely used one-photon brain imaging technique is confocal microscopy, which relies on a focused laser beam to elicit fluorescence in a single focused point, with a pinhole in the imaging path to reject out-of-focus fluorescence.^9^^–^^12^ As with two-photon microscopy, confocal microscopy techniques rely on scanning, which limits image acquisition speeds.

To scale the number of neurons that can be monitored simultaneously and capture rapid transient fluorescence events at millisecond speeds, it is necessary to consider scanless acquisition techniques where data are acquired in parallel, typically on millions of pixels with a camera sensor. Under these conditions, the acquisition speeds are now only limited by the data throughput of the camera, typically several gigabits per second.

The recent improvement of genetically encoded calcium and voltage indicators enables neural activity tracking with single-cell resolution and constantly improving temporal precision. Calcium indicators such as jGCaMP8^13^ and XCaMP-Gf^14^ enable millisecond-level half-rise times, which are 10 times faster than those of the widely used GCaMP6 calcium^15^ indicators, requiring over 100-Hz sampling speeds. Recently developed genetically encoded voltage indicators^16^^–^^21^ enable access to the membrane potential of neurons with millisecond temporal resolution and should be ideally used in combination with optical techniques that can achieve at least 200-Hz sampling speeds. With faster reporters, one bottleneck to performing large-scale imaging is the camera’s acquisition speed. Even with high-performance sCMOS cameras (e.g., Andor Zyla), sampling speeds are at or below 100 Hz with the full FOV. To perform voltage imaging recording with faster sampling rates, only a fraction of the camera’s sensors are used, limiting the number of neurons captured by the camera. To achieve higher acquisition speed without compromising spatial information, new microscope designs have been recently explored that computationally reassign each region of interest (ROI) in the FOV to a single pixel on a smaller detector^22^ and achieved neuronal voltage dynamics at over 5-KHz sampling rates. Remote refocusing light-sheet microscopy techniques that only require scanning along one axis have also been improved to enhance the volume scan rate^23^ and have achieved voltage dynamics recordings of whole brains in larval zebrafish at a volumetric rate of 200 Hz, albeit with increased device complexity.

To capture fast light intensity variation with a high temporal resolution, streak cameras^24^ have been developed and applied to fluorescence lifetime^25^ and phosphorescence lifetime^26^ imaging, but not to *in vivo* fluorescence imaging of the brain. The mechanical streak camera utilizes a rotating mirror or a moving slit system to deflect the light beam and generate a streak pattern on the camera sensor.^27^ However, its maximum scanning speed and temporal resolution are limited. For example, the maximum small angle bandwidth of Thorlabs galvo mirror GVS211 is 1 kHz. The optoelectronic streak camera, which rapidly changes the electric field applied to photoelectrons to obtain the streak pattern, achieves much better temporal resolution up to hundreds of femtoseconds.^28^ An alternative approach for fast imaging using rotating mirrors was introduced for single-molecule fluorescence spectroscopy.^29^ Instead of generating a streak pattern on the camera sensor, the authors swept the galvo mirror in separate steps resulting in equally spaced single-molecule spots on the image plane with higher temporal resolution.

Here, we developed a compressive fluorescence microscope that combines a mechanical streak camera with a digital micromirror device (DMD) to achieve sampling speeds that outpace the nominal frame rate of the camera. To reduce out-of-focus fluorescence and ensure spatial sparsity of ROIs, we performed targeted excitation with a digital micromirror device, illuminating only an ROI where a neuron was identified.^12^^,^^17^^,^^30^^–^^32^ As we only require a scanning speed of ∼40  Hz with a small scanning angle, we utilized a galvo mirror to controllably deflect light received along a line of camera pixels during the exposure of a single frame. We then digitally recovered high-speed fluorescence traces from the streak movie using ridge regression.^33^

In Sec. 2, we present the optical setup design and reconstruction algorithms for our compressive microscope. In Sec. 3, we show simulations and experimental results that demonstrate the capabilities of our microscope. We performed proof-of-concept simulations using a published voltage imaging dataset.^16^ We then showed experimental imaging of fluorescent beads and *in vivo* recordings of neurons in larval zebrafish. In Sec. 4, we summarize our findings and propose future goals.

## 2 Materials and Methods

### 2.1 Optical Setup

**Fig. 1:**
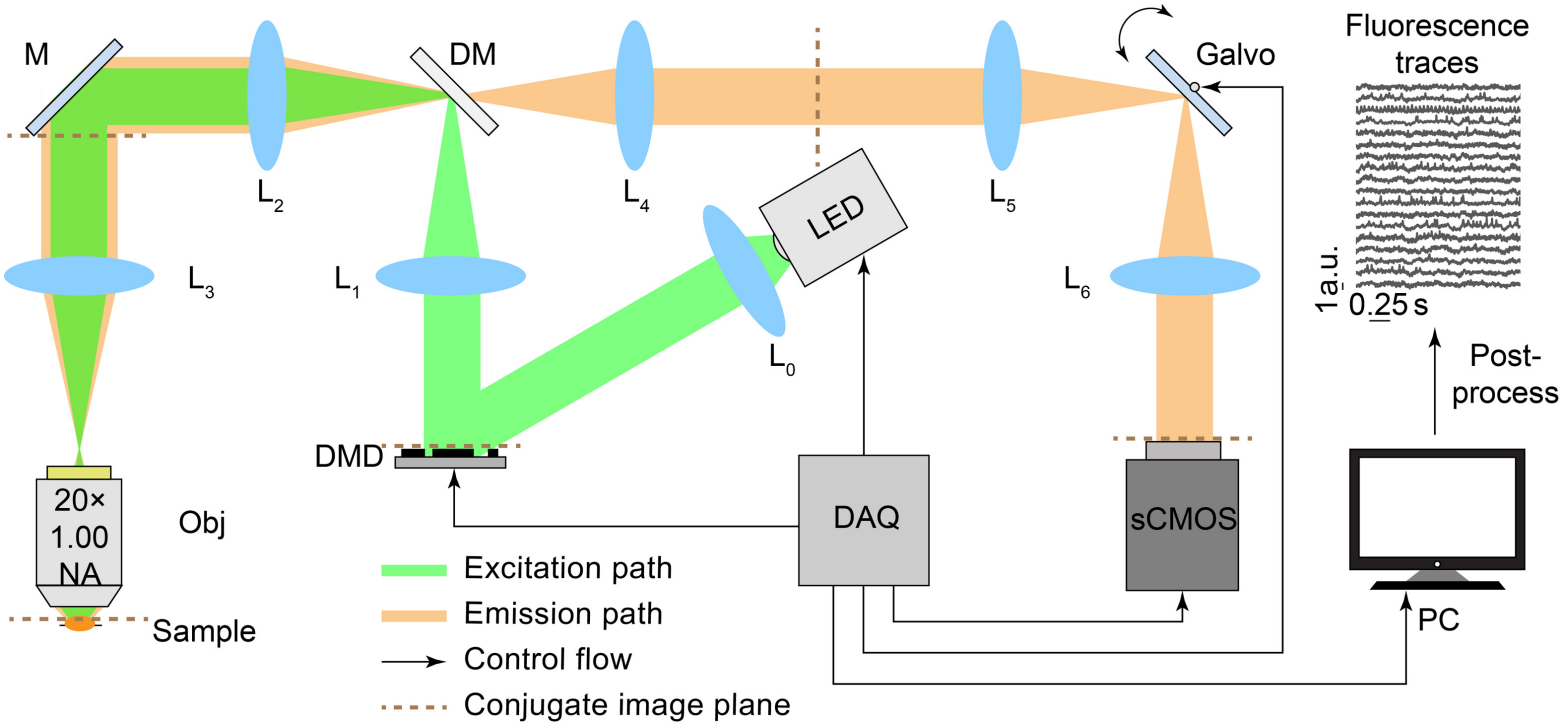
Experimental setup of the compressive fluorescence microscope. The excitation light (green path) is spatially modulated by the DMD to restrict illumination to the regions of interest. The emission light (orange path) is deflected by the galvanometer mirror to create a streak image on the camera. The galvanometer mirror and the camera acquisition trigger are digitally synchronized by the computer controlling the acquisition. Streak images are then digitally processed to reconstruct fluorescence traces with a higher effective sampling rate. (DM, dichroic mirror; M, mirror; Obj, objective; DAQ, data acquisition; L, lens).

Our microscope design is shown in Fig. 1. A DMD placed in the excitation path of a widefield fluorescence microscope performs selective fluorescence excitation with custom 2D patterns to reduce out-of-focus fluorescence and background noise. The digital light processing (DLP) DMD (DLP6500FLQ, Texas Instruments, Dallas, Texas, United States) is an array of 1920×1080 micromirrors that can be shifted between two positions oriented at +/−12 deg to either reflect light or redirect it toward a beam dump. Each of the DMD mirrors is independently configurable at frequencies up to 11,574 binary frames per second. We used a 20×, 1.00 NA water immersion microscope objective (XLUMPLFLN 20× Objective, Olympus, Tokyo, Japan). A galvo mirror (GVS211, Thorlabs, Newton, New Jersey, United States) is placed in the pupil plane of the imaging path and synchronized with the camera image trigger to distribute the light received by the sensor along one streak per frame. The camera (Andor Zyla 5.5 sCMOS camera, Oxford Instruments, Abingdon, United Kingdom) has 2560×2160 active pixels and offers frame rate performance up to 80 fps with the whole FOV under the global shutter. We chose the global shutter over a rolling shutter for recording because, under rolling shutter mode, streaks at different locations of the sensor might record different time points, posing unnecessary challenges for reconstruction (see Supplementary Material). The light source (LCS-0525-60-22 for the fluorescent bead experiments and LCS-0470-50-22 for the zebrafish experiments, Mightex, Toronto, Ontario, Canada), DMD, galvo mirror, and sCMOS camera are connected to a data acquisition system (DAQ, Ni PCIe 6363, National Instruments, Austin, Texas, United States) controlled by the computer. By capturing photons from the same neuron in different pixel locations along the streak direction, our microscope enables high-speed fluorescence activity reconstruction beyond the camera frame rate and minimizes photon loss without substantially lowering the signal-to-noise ratio (SNR) (see Supplementary Material for additional information about the lenses and optical properties of the setup).

### 2.2 Pipeline and Reconstruction Algorithms

**Fig. 2:**
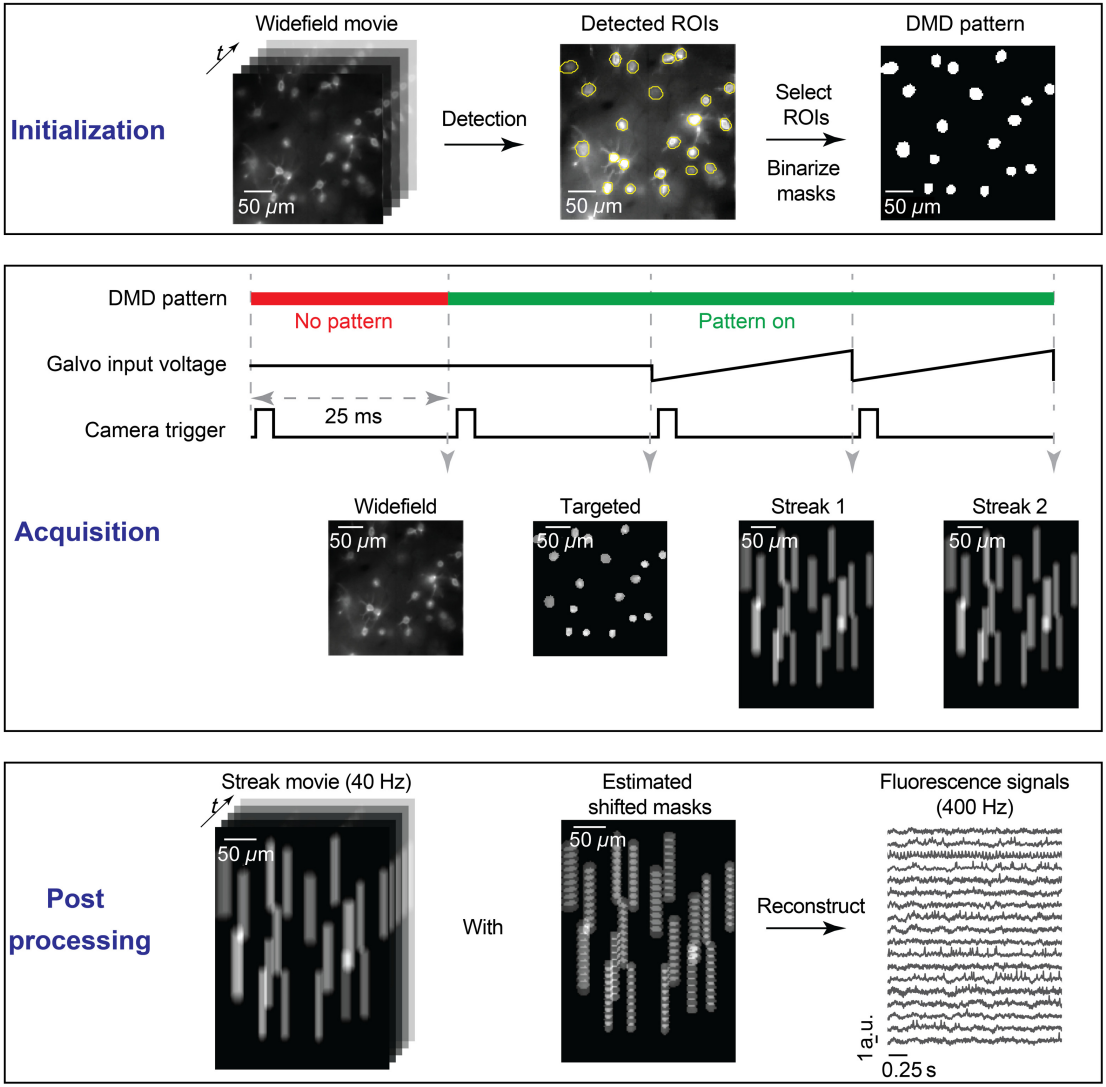
Pipeline of the compressive fluorescence microscope. In the initialization step, we identify binary masks for ROIs from a widefield fluorescence imaging movie. In the acquisition step, we use the DMD for targeted illumination and a galvo mirror to generate a streaked movie. In the postprocessing step, using the shifted masks of neurons, high temporal resolution fluorescence signals are digitally reconstructed from the streak movie.

The pipeline of our compressive microscope is shown in Fig. 2 and includes three steps: initialization, acquisition, and postprocessing. In the initialization step, we first map the coordinates of the camera to the coordinates of the DMD for accurate targeted illumination. We display a 14-by-14–point grid pattern on the DMD with known coordinates in the DMD space to selectively excite a fluorescence slide. The camera records a picture of the known fluorescence pattern. We then digitally find the local maxima corresponding to each illumination point in the system of coordinates of the image, as captured by the camera. We then fit two separate quadratic regression models using the x and y coordinates in the image space as predictor variables and the x (or y) coordinates in the DMD space as response variables. Given the fitted models, we can predict which DMD pixels must be selected to illuminate custom neurons identified by the camera during the experiment. After mapping spatial coordinates, we turn on all the DMD pixels and record a widefield movie (between 25 s and 1 min at the desired frame rate) of the sample. Next, in simulation, we apply a neural network method to identify a spatial mask associated with each ROI. For beads and zebrafish experiments, we find local maxima in the Gaussian-smoothed mean or standard deviation image to detect centers of beads or neurons and then generate circle masks using selected centers (see Sec. 3 for more details). Masks are combined and binarized for DMD display.

During the acquisition step, we first record a targeted illumination video with the DMD displaying the binarized mask pattern. The targeted illumination video is important for two reasons. First, the video is used to check the targeted illumination quality. If the optical setup is misaligned or if two ROIs are too close to one another (or even overlap), there might exist optical crosstalk across ROIs, i.e., nontargeted neurons or beads receive and emit light. By scrutinizing the targeted illumination video, we identify and remove ROIs that suffer from crosstalk in the postprocessing step. Second, we use the targeted illumination video to compute a weighted mask a(x,y) associated with each ROI using the nonnegative matrix factorization (NMF) algorithm. Weighted masks are used for reconstruction in the postprocessing step.

For reconstruction purposes, we also estimate relative shifts of ROIs under different galvo mirror input voltages. We drive the galvo mirror with a series of input voltages ranging from $-V_{\max}$ to $V_{\max}$ with equal intervals depending on the data compression ratio and capture shifted targeted illumination images. Next, for each shifted image, we compute its cross-correlation with the original targeted illumination image (when the galvo mirror input voltage is equal to 0), and the maximum cross-correlation indicates the relative shifts. We further estimate the subpixel shifts (e.g., 0.2 pixels shift on the x-axis) with bilinear interpolation. With computed shifts, we linearly translate the spatial footprints as our approximation of spatial footprints under different driving voltages for the galvo mirror, for each ROI. The above method is similar to estimating motion shifts in calcium imaging analysis.^34^

During the acquisition step, we drive the galvo mirror with a sawtooth wave that is synchronized and phase locked with the camera frame trigger at a frequency of $f_{\mathrm{cam}}$ Hz. In this configuration, each acquired frame experiences exactly one sweep of the galvo mirror, and the same sweeping motion occurs for every subsequent frame.

In the postprocessing step, we reconstruct fluorescence traces for each ROI at a virtual sampling rate of $f_{\mathrm{rec}}=r\times f_{\mathrm{cam}}\;\mathrm{Hz}$, where r is the data compression ratio. As the camera FOV for imaging experiments is large (2560 pixels by 2160 pixels), it is inefficient to analyze all ROIs at the same time with big, sparse masks. Instead, we extract a small region (200 pixels by 200 pixels in space) around every ROI, and we reconstruct signals from each ROI independently (except for simulations where FOV is small and all ROIs are postprocessed simultaneously). As ROIs are postprocessed independently in beads and zebrafish experiments, we avoid overlapping of nearby streaks to prevent signal crosstalk and ambiguities in the reconstruction of traces. For simplicity and illustrative purposes, we assume that the streak movie in the following paragraphs contains a single ROI.

For reconstruction, each streak movie s(x,y) of a single ROI can be viewed as the integration of the fluorescence activity c(t) along the trajectory (Δx(t),Δy(t)) of its spatial footprint a(x,y) across time period from 0 to T plus random noise ϵ(x,y)^35^

$$s(x,y)=\int_{0}^{T}a(x-\Delta x(t),y-\Delta y(t))c(t)dt+\epsilon (x,y).$$ (1)

Here, Δx(t) and Δy(t) are the shifts along the x-axis and y-axis at time point t, respectively. T is the exposure time for one streak frame.

The integral can be approximated by the sum of a set of fixed spatial footprints $a_{i}(x,y)=a(x-\Delta x(i\Delta t),y-\Delta y(i\Delta t))$, i=0,1,…,r−1, estimated in the acquisition step, multiplied by the fluorescence activity c(iΔt), where $\Delta t=\frac{T}{r}$ and the data compression ratio r decides the number of different fixed spatial footprints

$$s(x,y)=\overset{r}{\underset{i=1}{\sum }}a_{i}(x,y)c(i\Delta t)+\epsilon (x,y).$$ (2)

The above equation can be rewritten in a matrix format

s=Ac+ϵ, (3)

where $s\in R^{n}$, $A=[a_{1},a_{2},\ldots ,a_{r}]\in R^{n\times r}$, $c\in R^{r}$, $\epsilon \in R^{n}$ n is the number of pixels in one streak frame.

Given the streak frame s and system matrix A, we compute fluorescence traces c. When shifted spatial masks $a_{i}$ are close to one another, the problem turns out to be an ill-posed deconvolution problem that cannot be solved with linear regression.^36^ Adding regularization is a typical way to solve an ill-posed inverse problem.^37^ Here, we add the $L_{2}$ norm of traces c (also known as Tikhonov regularization) and solve a ridge regression problem^33^

$$\underset{c}{\min}\;\frac{1}{2}{\left\|s-Ac\right\|}_{2}^{2}+\alpha {\left\|c\right\|}_{2}^{2},$$ (4)

where α is the regularization strength. When α→∞, the result of ridge regression is similar to weighted averaging. When α→0, the result is the same as linear regression. The pick of the regularization strength α influences the performance of ridge regression greatly. We found that using cross-validation with a negative mean square or R-squared as a metric often underestimated the regularization strength. Instead, we selected the regularization strength α based on the spectral condition number^38^ 0pt. The spectral condition number of the matrix $\left(A^{T}A+\alpha I\right)$ is equal to $\frac{\lambda_{\max}}{\lambda_{\min}}$ where $\lambda_{\max}$ and $\lambda_{\min}$ were the largest and smallest eigenvalues of the matrix $\left(A^{T}A+\alpha I\right)$, respectively. When α→0, the condition number could be large if there is much overlapping. When α→∞, the condition number is close to 1. Before performing ridge regression, we first computed the condition number of the matrix $\left(A^{T}A+\alpha I\right)$ with different α from the list $\left[10^{-4},10^{-3},10^{-2},10^{-1},1,10,10^{2},10^{3},10^{4}\right]$. Next, we set the threshold value as 10 and picked the smallest α such that the condition number was less than the threshold. We tested and kept the threshold the same across all simulations/experiments across the paper. The reader should expect reasonable performance with this threshold given similar imaging conditions. After determining the regularization strength α, we solved the ridge regression problem using the solver implemented in the scikit-learn^39^ package.

In simulations and fluorescent beads experiments, we compared the ridge regression method with two other methods: weighted averaging and NMF. The weighted averaging method performs a weighted average of pixel values inside each spatial mask a

$$c=A^{T}s.$$ (5)

The method is simple and fast but does not consider demixing signals from different overlapping spatial footprints. The other method is NMF, which can be formulated as follows:

$$\underset{A\geq 0,C\geq 0}{\min}\;{\left\|S-AC\right\|}_{F}^{2},$$ (6)

where $S=[s_{1},s_{2},\ldots ,s_{m}]\in R^{n\times m}$ is the streak movie of m frames, $C=[c_{1},c_{2},\ldots ,c_{m}]\in R^{r\times m}$ is the extracted fluorescence traces, and F means the Frobenius norm. NMF requires every element in matrices A and C to be nonnegative. Unlike ridge regression and weighted averaging, which deal with each streak frame s independently and do not change spatial masks A, the NMF method optimizes both spatial masks A and temporal traces c. We are interested to see if optimizing the spatial masks A will improve reconstruction performance. The NMF problem is solved with the hierarchical alternating least square algorithm.^40^

As more photons are collected at the center of the streak than at the two ends of the streaks, traces reconstructed from different shifted spatial masks might be not on the same scale. To remedy this, we perform z-score standardization on fluorescence signals from each spatial mask independently (corresponding to each row of the matrix C) and concatenate each column of the matrix C to reconstruct the fluorescence traces at high temporal resolution. We perform z-score standardization again on the reconstructed traces and compare them with the reference fluorescence traces, if available.

### 2.3 Experiments in Zebrafish

For all experiments, we used 6-day old larval zebrafish from a Casper (nacre -/-, roy -/-) background; nacre -/- mutants lack melanocytes, which lead to skin pigmentation, but retain eye pigmentation, whereas roy -/- mutants have translucent skin, lack iridophores, another source of pigmentation, and have uniformly pigmented eyes.^41^ Together, these mutations enable high-quality brain imaging without compromising visual function. Adult zebrafish were maintained on a 14-h light/10-h dark cycle, and fertilized eggs were collected and raised at 28.5°C. Embryos were kept in E3 solution (5-mM NaCl, 0.17-mM KCl, 0.33-mM ${\mathrm{CaCl}}_{2}$, 0.33-mM ${\mathrm{MgSO}}_{4}$). All experiments were approved by Duke University’s standing committee on the use of animals in research and training. We performed all imaging experiments on transgenic zebrafish expressing nuclear-targeted GCaMP6s (elavl3:h2b-GCaMP6s). Zebrafish were paralyzed to prevent movement from disrupting compressive imaging. We placed fish in a droplet of $1\;\mathrm{mg}.{\mathrm{ml}}^{-1}$ α-bungarotoxin (Sigma-Aldrich Burlington, Massachusetts, United States) for a duration of 110 s before returning them to egg water immediately prior to embedding them in 2% agarose. Zebrafish were imaged under a 470-nm LED light source with an intensity of 8 $4.2\;\mathrm{mW}\cdot {\mathrm{mm}}^{-2}$ (LED driver voltage 0.3 V) at the focal plane with the objective of widefield imaging. The intensity was $65.4\;\mathrm{mW}\cdot {\mathrm{mm}}^{-2}$ (LED driver voltage 10 V) for targeted illumination and streaking imaging. In all cases, we estimated the intensity by calculating the total energy under the objective divided by the estimated illumination area ($∼0.26\;{\mathrm{mm}}^{2}$) with all DMD pixels on.

## 3 Results

### 3.1 Simulation Results

**Fig. 3:**
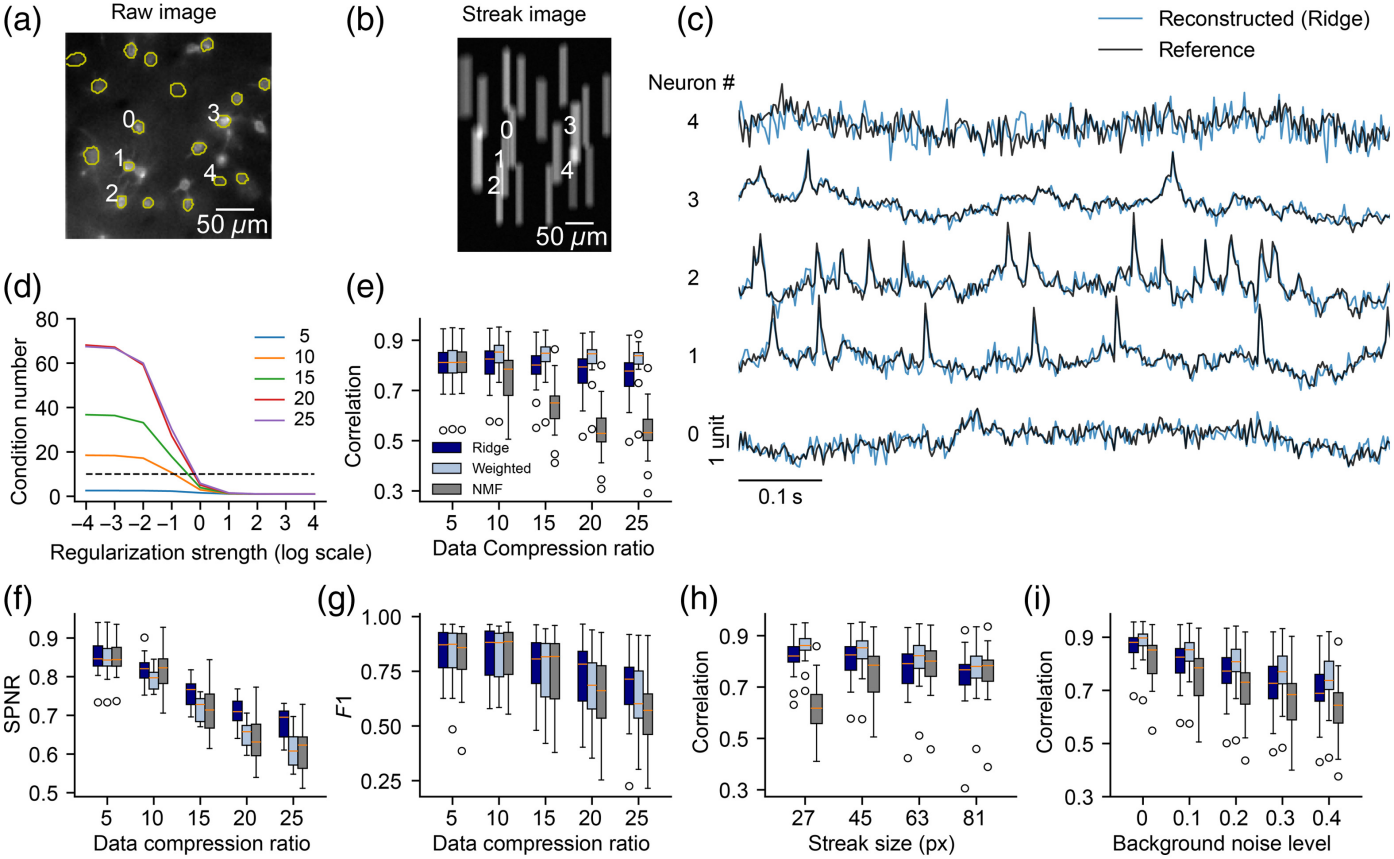
Reconstruction of simulated voltage imaging data recorded from mouse L1 cortex. (a) Raw image of voltage imaging data overlaid with detected ROIs (yellow). Traces from four selected neurons were shown in panel (c). (b) A streak image from the streak video. Traces from selected neurons were shown in panel (c). (c) Examples of reconstructed (blue) and reference (black) voltage fluorescence traces (z-score). (d) Condition number with different regularization strength ($\log_{10}\;\alpha$) at 5, 10, 15, 20, and 25 data compression ratios. The threshold is set as 10 for picking regularization strength α (dashed line). (e)–(g) We computed three metrics, including correlation, F1 score, and relative spike-to-noise ratio (SPNR), to measure the reconstruction quality at 5, 10, 15, 20, and 25 data compression ratios. Three reconstruction methods were tested, including ridge regression (navy), weighted averaging (light blue), and nonnegative matrix factorization (gray). (h) Correlation with 27, 45, 63, and 81 pixels streak size. (h) Correlation with 0, 0.1, 0.2, 0.3, and 0.4 background noise level. (e)–(i) Each point represents the result of one neuron [middle line (orange) median, box the first quartile and the third quartile, whiskers extended from the box by the 1.5× interquartile range].

To validate whether our compressive microscope was feasible, we generated simulated data and tested the reconstruction performance. The raw voltage imaging data [20,000 frames × 128  pixels×128  pixels, Fig. 3(a)] were collected from the mouse L1 cortex using Voltron indicator recording at a frame rate of 400 Hz.^16^ We used Mask R-CNN,^42^^,^^43^ a convolutional neural network, to detect ROIs that were ∼10  pixels in diameter in the raw movie. We removed ROIs, the centroids of which were within 4 pixels away from other centroids on the x-axis or 30 pixels away from other centroids on the y-axis (streak direction). We linearly interpolated the raw data for each pixel across time and simulated targeted illumination by masking pixels outside the ROIs. To simulate background noise in the targeted illumination video, we added an independent and identically distributed Gaussian noise to each pixel in every frame. The mean and variance of the noise were equal to the average background noise in the raw video multiplied by the background noise level (default 0.1) to control the intensity of the background noise. Next, we simulated the galvo mirror scanning by linearly translating each frame along the y-axis (see the streak movie in Fig. 2). We summed every r (equal to data compression ratio, default is 10) translated frames to generate a streak video of a 40-Hz frame rate [Fig. 3(b)] and added the detector noise from a Gaussian distribution with its mean and variance equal to that pixel’s intensity independently to each pixel. Traces were reconstructed using three methods: ridge regression, weighted averaging, and NMF. Extracted traces were compared with the reference traces computed by averaging pixel values inside each ROI in the raw movie.

Examples of reconstructed traces using ridge regression were compared with reference traces [Fig. 3(c)]. We computed the condition number of the matrix $\left(A^{T}A+\alpha I\right)$ with different regularization strengths ($\log_{10}\;\alpha$) for data compression ratio values of 5, 10, 15, 20, and 25 [Fig. 3(d)]. We set 10 as the threshold and picked the smallest α such that the condition number was less than the threshold. We used VolPy ^43^ to extract spikes from the reconstructed and reference traces. We computed the Pearson’s correlation coefficients between reconstructed traces and reference traces [Fig. 3(e)], relative spike-to-noise ratio [SPNR, measures the average spike height of reconstructed traces over the average spike height of reference traces, Fig. 3(f)], and F1 scores [which measures spike detection accuracy between reconstructed traces and reference traces, Fig. 3(g)] with 5, 10, 15, 20, and 25 data compression ratio at a fixed streak size of 45 pixels. Traces from all neurons were computed for correlation coefficients (n=18), whereas only active neurons with more than 50 spikes detected in reference traces were selected and computed for SPNR and F1 score (n=13). With increasing data compression ratio, reconstruction performance was worse when measured with SPNR and F1 score, but not necessarily when measured with correlation. Reconstruction performance of ridge regression was better than that of NMF across all three metrics. Weighted averaging achieved a lower SPNR when compared with that of the ridge regression likely because the weighted averaging method did not demix signals from nearby spatial footprints. Next, we measured the correlation between reconstructed and reference traces at a data compression ratio of 10 under different streak sizes [Fig. 3(h)]. A bit surprisingly, the performance of ridge regression and weighted averaging decreased a little with increasing streak size, which might be because fewer photons were on each camera pixel resulting in an increase in detector noise. Finally, we tested correlation under different background noise levels [Fig. 3(i)]. As expected, with increasing background noise on the targeted illumination video, it was harder to reconstruct traces accurately.

During simulations, we found that the following set of parameters worked well for reconstruction: camera rate 40 Hz, data compression ratio 10, streak size 45 pixels, and background noise level 0.1.

### 3.2 Imaging Fluorescent Beads

**Fig. 4:**
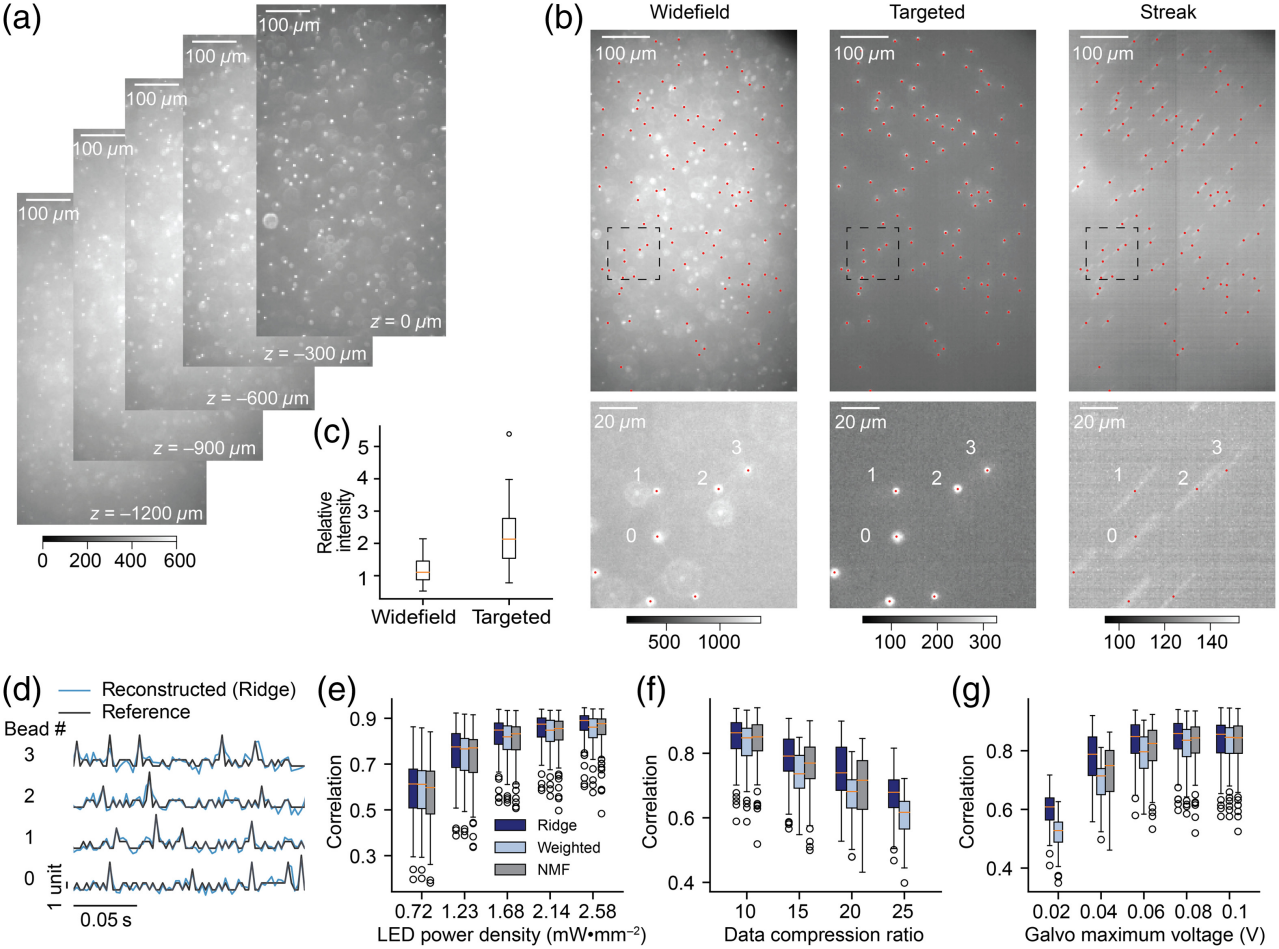
Imaging fluorescent beads with our compressive streak microscope. (a) 2D static images of the sample at different depths. The color bar showed the range of intensity for all widefield images. (b) static fluorescence images of a suspension of fluorescent beads with widefield (left) and targeted (middle) illumination at a depth equal to −600  μm. The mean image of the streak movie (Video 1, MP4, 9.02 MB [URL: https://doi.org/10.1117/1.NPh.12.2.025013.s1]) is shown on the right. Selected beads for targeted illumination and reconstruction were labeled with red dots. Images inside black dashed squares were magnified below. Traces from selected beads were shown in panel (d). Color bars showed the range of intensity for widefield and targeted and streak images under the same imaging power density. Note the exposure time in panel (b) was different from that shown in panel (a). (c) For beads with widefield and targeted illumination, we computed relative fluorescence intensity equal to the maximum fluorescence of the bead subtracted from the background fluorescence and then divided by the background fluorescence. (d) Examples of reconstructed traces (400 Hz) and reference traces (z-score) from selected beads. (e) Correlation between reconstructed traces and reference traces with different LED power densities, including 0.72, 1.23, 1.68, 2.14, and $2.58\;\mathrm{mW}\cdot {\mathrm{mm}}^{-2}$ (LED driver voltages corresponding to 0.25, 0.5, 0.75, 1.0, and 1.25 V). Three reconstruction methods were tested, including ridge regression (navy), weighted averaging (light blue), and NMF (gray). (f) Correlation between reconstructed traces and reference traces with a series of data compression ratios, including 10, 15, 20, and 25. (g) Correlation between reconstructed traces and reference traces with different galvo maximum voltages, including 0.02, 0.04, 0.06, 0.08, and 0.1 V. (e)–(g) Each point represents the result of one bead [middle line (orange) median, box the first quartile and the third quartile, whiskers extended from the box by the 1.5× interquartile range]. N=101 across all experiments.

To evaluate the microscope’s performance under experimental conditions, we built the optical setup and imaged fluorescent beads (Fig. 4). A green LED (LCS-0525-60-22, Mightex, Toronto, Ontario, Canada) was used as the excitation light source, and a filter set compatible with the LED was used for the dichroic mirror (49014 ET-mKO/mOrange, Chroma, Taoyuan City, Taiwan). Our sample is a static suspension (100  μL) of orange fluorescent beads (L9529, Sigma-Aldrich, 2-μm mean size) in a 1.2-mm-thick slice of polydimethylsiloxane (10 g).

We first recorded multiple 2D snapshots of the sample along the vertical (z) axis [Fig. 4(a)] and selected the z=−600  μm plane to be the focal plane [Fig. 4(b) left panel]. As in-focus beads were bright and easy to identify, we detected the centers of beads by computing the local maxima in the Gaussian-smoothed image, and we removed the maxima of out-of-focus beads that appeared as larger round objects or exhibited rings around their centers. Next, we displayed a pattern of filled circles with the locations of maxima as centers (radius equal to 15 pixels) on the DMD for targeted illumination [Fig. 4(b) mid panel]. We found that although beads were less bright in the targeted illumination image [see color bars in Fig. 4(b)], background fluorescence due to out-of-focus fluorescent beads was also greatly reduced. In both widefield and targeted illumination images, for each bead, we computed the signal-to-background ratio [Fig. 4(c)], which was equal to the maximum fluorescence of the bead subtracted from the background fluorescence and then divided by the background fluorescence. We observed that beads showed higher relative fluorescence intensity in the targeted illumination image.

Next, we implemented a linear streak by driving the galvo mirror with a sawtooth control voltage ranging from –0.08 to 0.08 V, and we captured streaked videos at a rate of 40 Hz with a data compression ratio of 10 (to target a virtual sampling rate of 400 Hz) [Fig. 4(b) right panel, Video 1, MP4, 9 MB]. To mimic fluorescence activity changes in beads, we rapidly switched patterns across 5 DMD subframes for every single virtual frame (2.5 ms under 400-Hz virtual sampling rate). To be more specific, we uploaded 390 different 1-bit subframes (390/5=78 virtual frames) to the DLP LightCrafter DLPC900 graphic user interface (GUI) (version 5.1.0) software (maximum 9 memory is 400 1-bit frames). During the experiment, the DMD displayed all uploaded subframes one by one with equal time duration (0.5 ms) controlled by a dedicated trigger from the National Instruments data acquisition (NiDAQ) device. It repeated the sequence of the patterns from the beginning to the end of each cycle. To mimic action potentials, for each bead independently, we randomly selected 5 out of 78 virtual frames (not DMD subframe) when we turned the DMD on for 3, 4, or 5 subframes out of 5 subframes, equivalent to a 5/0.195=26  Hz firing rate. For the remaining 73 virtual frames, we mimicked the baseline fluorescence fluctuations by turning the DMD on for 0 or 1 subframe out of 5 subframes. As each bead fired at randomly selected virtual frames, the correlation among beads was close to 0. We used the number of subframes that the DMD is on for every single virtual frame as the reference signals for each bead. As described in Sec. 2, we estimated weighted masks for each bead and relative shifts of beads’ locations at different galvo mirror input voltages. Finally, we reconstructed fluorescence traces with three algorithms for each bead.

Examples of reconstructed traces using ridge regression from selected beads shown in Fig. 4(b) were compared with the corresponding reference traces in Fig. 4(d). We computed the correlation between reconstruction traces and reference traces under different conditions. We first tested the correlation with different LED power densities [Fig. 4(e)]. As expected, the compressive microscope performed better at higher LED power densities because more photons were collected by the camera resulting in an increase in the SNR of the streak movie. Next, we tested correlation under different data compression ratios [Fig. 4(f)] with the galvo maximum voltage fixed to 0.08 V. We found that the correlation decreased with higher data compression ratios. When the data compression ratio increased to 25, the NMF method failed likely because the streak length was too short for reconstructing with high data compression ratios. Finally, we tested the correlation between reconstructed traces and reference traces under different galvo maximum voltages from 0.02 to 0.1 V with a fixed data compression ratio of 10 [Fig. 4(g)]. We found that as the galvo maximum voltage increased, correlation increased. When the galvo maximum voltage was equal to 0.02 V, the NMF algorithm failed, showing its instability when the streaks had small trajectories. Across these tests, the ridge regression method showed better performance against weighted averaging and NMF. It was also more robust than NMF when the streak size was small or the data compression ratio was high.

During fluorescent beads experiments, we found that the following set of parameters worked well for experiments and reconstruction: DMD circle radius 15 pixels, camera rate 40 Hz, data compression ratio 10, LED power density $2.14\;\mathrm{mW}\cdot {\mathrm{mm}}^{-2}$, and galvo maximum voltage 0.08 V.

### 3.3 *In vivo* Imaging of Neurons in Zebrafish

**Fig. 5:**
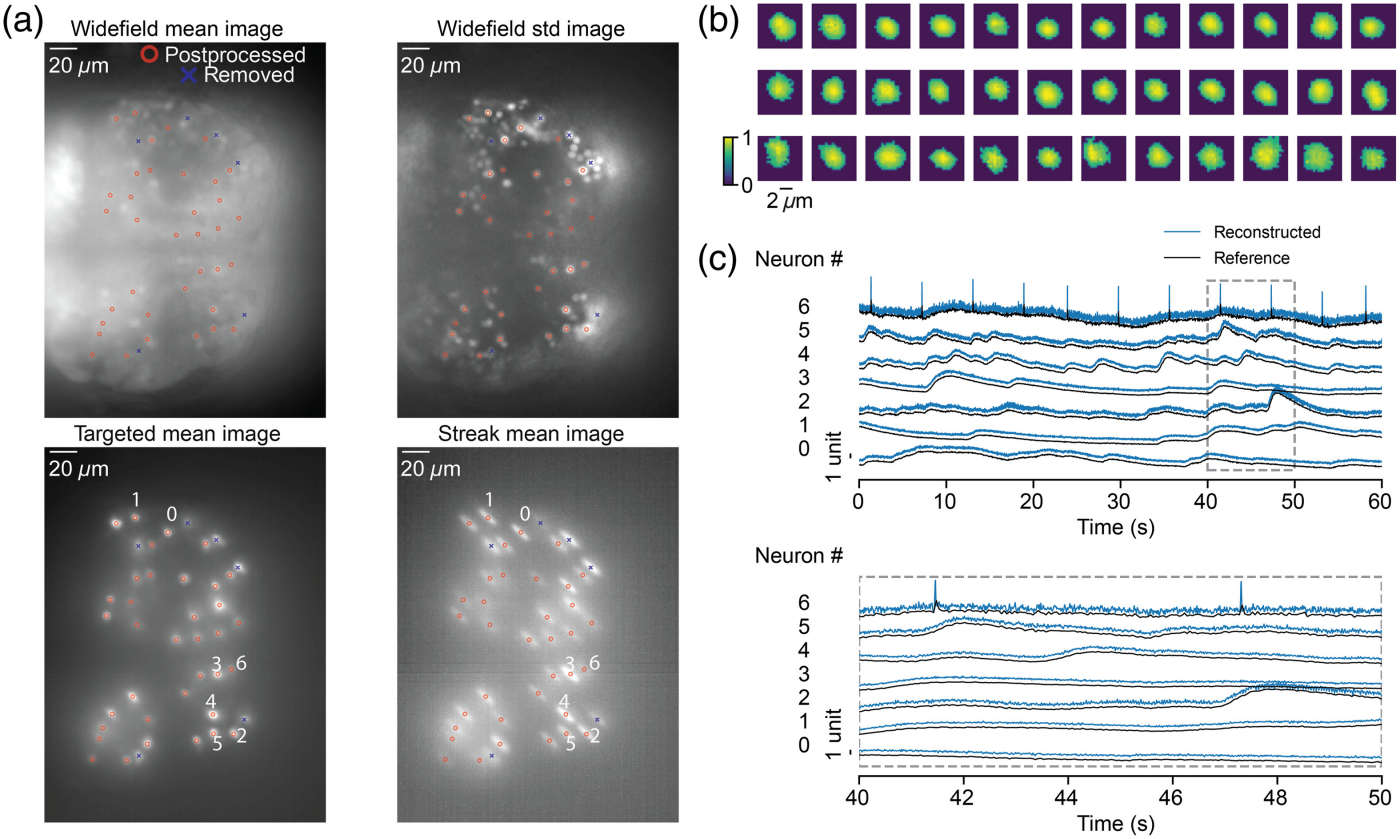
Fluorescence imaging of neural activity in zebrafish at 200 Hz. (a) The mean image of the widefield movie (top left; Video 2, MP4, 8.55 MB [URL: https://doi.org/10.1117/1.NPh.12.2.025013.s2]), the standard deviation image (top right) of the widefield movie, the mean image of the targeted illumination movie (bottom left; Video 3, MP4, 8.69 MB [URL: https://doi.org/10.1117/1.NPh.12.2.025013.s3]), and the mean image of the streak movie (bottom right; Video 4, MP4, 8.05 MB [URL: https://doi.org/10.1117/1.NPh.12.2.025013.s4]) are shown. 36 Neurons labeled with red circles were selected for reconstruction. Neurons that suffered from optical crosstalk in targeted illumination movies were labeled with blue crosses and were not postprocessed. (b) Thirty-six spatial footprints of neurons computed from targeted illumination video used for reconstruction. (c) Reconstructed fluorescence signals (blue, z-score, 200 Hz) computed from a 1-min-long streak movie (20 Hz) across seven selected neurons. Reference traces (black, z-score, 20 Hz) were computed by averaging signals in each streak for each frame. Traces inside dashed rectangles were magnified on the bottom.

To validate the performance of the compressive microscope in living animals, we performed calcium imaging of neural activity in larval zebrafish (Fig. 5). A blue LED (LCS-0470-50-22, Mightex, Toronto, Ontario, Canada) served as the light source, and a dichroic mirror with an appropriate filter set was utilized (49002 ET-EGFP, Chroma, Taoyuan City, Taiwan). To reduce scattering, we performed targeted illumination and streak imaging on dorsal habenula neurons with an 800×600  pixels FOV (289×217  μm), which presented higher SNR in the widefield movie in comparison to SNRs of neurons from other regions in zebrafish, rendering it suitable for compressive imaging.

We first captured a 1-min-long widefield video of the zebrafish [Fig. 5(a) top left and top right panels; Video 2, MP4, 8.5 MB] recording at 5 Hz, generating a standard deviation image by computing the standard deviation of the signal for each pixel over time. Next, we filtered this standard deviation image with a Gaussian kernel of size 1 to detect centers of neurons by finding the local intensity maxima. As neurons were densely distributed across the zebrafish brain, we selected only a subset of these centers for targeted illumination to prevent possible overlapping in the streak movie. To include as many neurons as possible for streak imaging, we selected centers sequentially based on values of local maximum and excluded centers that were either (1) within a 55-pixel distance from any selected centers along the streak direction or (2) within a 20-pixel distance from any selected centers perpendicular to the streak direction.

Next, we generated an image of filled circles using selected centers (radius equal to 5 pixels) and displayed it on the DMD. Given that the zebrafish neurons were dimmer than the fluorescent beads, we increased the LED driver input to maximum voltage (10 V, or a power density of $65.4\;\mathrm{mW}\cdot {\mathrm{mm}}^{-2}$) for better SNR when performing targeted illumination and streak imaging. During the experiment, we did not observe significant photobleaching. We recorded a 1-min-long targeted illumination movie recording at 5 Hz [Fig. 5(a) bottom left panel; Video 3, MP4, 8.7 MB]. By examining the targeted illumination movie carefully during the postprocessing, we manually removed neurons that suffered from crosstalk [Fig. 5(a), blue crosses]. Although further decreasing the size of the patterned circles reduced the probability of crosstalk, too few emission photons resulted in poor imaging quality. From the targeted illumination movie, we extracted spatial footprints [Fig. 5(b)] for each neuron.

Finally, we controlled the galvo mirror with a sawtooth driving voltage ranging from –0.04 to 0.04 V at 20 Hz, synchronized with the camera with one sawtooth pattern per frame, and we recorded a 1-min-long streak movie, with a reconstruction for a data compression ratio of 10 [Fig. 5(a) bottom right panel; Video 4, MP4, 8 MB]. We limited the streak size to increase the number of photons in each pixel while still being able to reconstruct the high temporal resolution traces with precision. We performed ridge regression using footprints extracted from the targeted illumination video and applied Gaussian smoothing with a kernel size of 1 to the reconstructed signals to remove additional noise. The reconstructed traces (200 Hz) are shown in Fig. 5(c). We computed reference traces by averaging the signals over each streak for each frame. We did observe more fluctuations in the reconstructed signal compared with the reference signal. Besides the calcium dynamics, the compressive microscope captured small high-frequency peaks due to the camera noise in neuron number 6, whereas the high-frequency peaks in the reference traces showed much smaller amplitude and larger full width at half maximum. We also repeated the experiment and recorded a 1-min-long streak movie at 40 Hz, also with a data compression ratio of 10 (Supplementary Material; Video 5, MP4, 7.69 MB [URL: https://doi.org/10.1117/1.NPh.12.2.025013.s5]). Although the recorded streak movie showed higher levels of noise compared with the 20-Hz streak movie, we were able to reconstruct high temporal resolution fluorescence traces at 400 Hz.

## 4 Discussion and Conclusion

We developed a novel compressive microscope that addresses a growing need for faster fluorescence activity recording techniques driven by ongoing developments of faster fluorescent reporters of neural activity. By trading off local spatial information for better temporal resolution, we demonstrated the ability to record fluorescence activity at 200- to 400-Hz outpacing the nominal frame rates of our camera, with validation in fluorescence beads and *in vivo* experiments in larval zebrafish. When imaging brain regions with dense neural populations, we leveraged the optical patterning capabilities of a DMD to target neurons and achieve higher SNR selectively.

Perhaps the main performance limitation in our experimental setup is optical scattering in the zebrafish heterogeneous tissue. Scattering introduces noise near the targeted illumination areas [see Fig. 5(a) bottom left panel]. This noise adds up in the streak images reducing the streak imaging quality and lowering the reconstruction accuracy. Scattering is a well-known issue in bioimaging and is more noticeable in the zebrafish experiments where neurons are densely packed, than in the fluorescent beads experiments, where beads are sparsely distributed across the FOV. Increasing LED power does not mitigate scattering because it also increases the background noise.

Another limitation of our technique is that it requires sparsity of the neuronal population to trade spatial resolution for temporal resolution. In practice, targeted illumination with the DMD enforces sparsity when neurons are too densely clustered. In zebrafish experiments, we found that nearby untargeted neurons might be illuminated unintentionally due to scattering. If we further decrease the targeted illumination size, fluorescence signals will show lower SNR as fewer photons are received. On the other hand, if we further increase the targeted illumination size, there might be more overlapping neurons in the targeted illumination movie.

With streak microscopy, our goal is not to reconstruct sharp images but to monitor neural activity in large neuronal ensembles. The standard resolution criteria of fluorescence microscopy are not relevant to our application. Instead, we find that sparse excitation, which is needed to create discernible streaks, is also beneficial for mitigating the effects of scattering. By placing neurons of interest apart from each other, our technique enables sampling depths where fluorescence microscopy only yields blurry images.

Future work will aim to demonstrate large-scale neural activity tracking in zebrafish expressing voltage indicators. Overall, our microscope design leverages computational methods to improve the acquisition of sparsely encoded fluorescent information and digitally reconstruct data that are most valuable for experiential neuroscientists. The scalability of our design makes our approach complementary to the capabilities of the current state of the art and shows promising potential for large neuronal ensemble imaging tasks that multiphoton techniques are too slow to achieve.

## Supplementary Material

10.1117/1.NPh.12.2.025013.s01

10.1117/1.NPh.12.2.025013.s1

10.1117/1.NPh.12.2.025013.s2

10.1117/1.NPh.12.2.025013.s3

10.1117/1.NPh.12.2.025013.s4

10.1117/1.NPh.12.2.025013.s5

## Data Availability

Simulation, instrument control, and data analysis code are available at https://github.com/caichangjia/compressive_streak_microscopy. Experimental data presented in this article is publicly available in Zenodo. Raw voltage imaging data used for simulation can be found at https://zenodo.org/records/4515768.^44^
