# Supplementary material for: Compressive streak microscopy for fast sampling of fluorescent reporters of neural activity
Source: Neurophotonics. 2025 May 22;12(2):025013. doi: 10.1117/1.NPh.12.2.025013 (PMC12097808; doi:10.1117/1.NPh.12.2.025013)
Supplement: Supplementary file 1 [file NPh_012_025013_SD001.pdf]

Compressive streak microscopy for fast sampling of fluorescent reporters of neural activity: supplemental material

Changjia Cai[a, b], Owen Traubert[c, d], Jovan Tormes-Vaquerano[a], M.Hossein Eybposh[a, b], Srinivas C. Turaga[e], Jose Rodriguez-Romaguera[f, g, h], Eva A. Naumann[c, d], Nicolas C. Pégard[a, b, g, h, *]
[a]University of North Carolina at Chapel Hill, Department of Applied Physical Sciences, Chapel Hill, NC, USA, 27599
[b]University of North Carolina at Chapel Hill, Joint Department of Biomedical Engineering, Chapel Hill, NC, USA, 27599
[c]Duke University, Department of Biomedical Engineering, Durham, NC, USA, 27708
[d]Duke University, Department of Neurobiology, Durham, NC, USA, 27708
[e]HHMI Janelia Research Campus, Ashburn, VA, USA, 20147
[f]University of North Carolina at Chapel Hill, Department of Psychiatry, Chapel Hill, NC, USA, 27599
[g]University of North Carolina at Chapel Hill, UNC Neuroscience Center, Chapel Hill, NC, USA, 27599
[h]University of North Carolina at Chapel Hill, Carolina Stress Initiative, Chapel Hill, NC, USA, 27599

1 Compressive microscope design parameter

Here, we provide additional design parameters for the optical setup (See Figure 1). The LED emitter is first collimated by an integrated lens, L_0, which is included in the LED collimator source (LCS-0525-60-22 and LCS-0470-50-22, Mightex). We used 2 inches achromatic doublets as relay lenses L_1, L_2, and L_4 ($f = 150mm$, AC-508-150-A-ML, Thorlabs) and the tube lens L_3 ($f = 200mm$, AC-508-200-A-ML, Thorlabs). To minimize optical aberrations, we used a pair of camera lenses L_5 and L_6 ($f = 50mm$, $f/1.4D$ AF NIKKOR, Nikon) that relay the image on the sCMOS camera.

The full Field of View (FOV) was 720 $\times$ 853 μm^2, the FOV accessible by the DMD is 423 $\times$ 744 μm^2. These parameters were chosen so that the image of DMD would fit entirely within the camera's FOV, but not be significantly smaller; the imaging beam diameter at the galvo mirror was designed to be smaller than the maximal beam diameter that would fit within the galvo mirror (10 mm). Selecting a smaller DMD image at the camera, can enhance the resolution of the targeted illumination, but at the expense of a smaller FOV. The galvo mirror was placed in the Fourier (pupil) plane to deflect the light in the angular domain. We opted for this configuration to project rigid, minimally distorted, streak patterns on the camera sensor.

2 Analysis of compressive microscope design

2.1 *Trade-off between various parameters for streak imaging*

In the following analysis, we assume that each target is a circle with a radius of c pixels. There are n targets in the FOV with a density of p targets$\cdot$ pixels^{-2}. The streak length is equal to l pixels. For each streak, the distance between neighboring spatial footprints for reconstruction (e.g. a_i and a_{i+1} is equal to d pixels. The FOV has a size of s pixels^2. The compression ratio is r. For simplicity, we do not consider border effects.

We first acknowledge that the use of a DMD will generally restrict the FOV because the camera sensors and the DMD may have different aspect ratio and cannot be perfectly overlapping. While the full camera FOV is 2560 $\times$ 2160 pixels^2, the region that can be illuminated with custom

patterning on the DMD is restricted to a 2232×1270 pixels^2 area which corresponds to the DMD's FOV.

The theoretical upper limit for the number of targets, n_{max}, that can be individually addressed is proportional to the field of view size s and inversely proportional to the target radius c and the streak length l. $n_{max} \simeq \frac{s}{2cl} = 2835$ targets given $l = 50$ pixels, $c = 10$ pixels. The maximum density of targets $p_{max} = n_{max}/s \simeq \frac{1}{2cl}$. However, the above estimation of n_{max} and p_{max} is an upper bound for our experiments, because randomly distributed beads and zebrafish neurons are not evenly distributed in a two-dimensional grid arrangement. In zebrafish, neurons cluster to form dense groups. In practice, for experiments in the zebrafish habenula region, we were able to reliably image 36 neurons in a FOV of 600×800 pixels^2. Assuming the density of neurons remains similar across the FOV, the maximum number of neurons captured in a FOV of 2232×1270 pixels^2 should be close to $36 \times \frac{2232 \times 1270}{600 \times 800} = 213$ neurons.

Streak length, l, compression ratio, r, target radius, c, and the characteristic distance between adjacent spatial footprints, d, are related to each other following: $l = (r-1) \times d + 2c$. When $d > 2c$, the streak will be unnecessarily long, losing photons that are needed for digital reconstruction. For our experiments, to achieve an acceptable image reconstruction quality, the streak length needed to be at least $l = 52$ pixels on the camera sensor, when $c = 5$ pixels, which corresponds to a 0.04 V driving voltage amplitude for the galvo mirror.

The direction of the streak does not significantly affect our ability to reconstruct enhanced temporal sampling, except near the boundary of the FOV. The targets are preferred to be point-like or rounded shape. If the method is applied to dendrites/axons, then the direction of dendrites or axons needs to be considered together with the streaking direction. The reconstruction result is expected to be better when the direction of the dendrite/axon is perpendicular to the streak direction, and minimizes self-overlap in the streaked image.

2.2 *Photon collection efficiency*

Adding additional lenses/optical elements reduces the photon collection efficiency of the optical setup, because of imperfections in the alignment, and unwanted reflections at the optical interface of the lenses. The theoretical transmission of the achromatic doublet lenses we used is above 95% between 500 nm to 700 nm wavelength, according to the manufacturer data. As a result, using two of these lenses to physically relay the image (e.g. with lenses L_2, and L_4) is possible, with a loss in photon collection efficiency that is at most 10%.

To minimize optical aberrations, instead of using achromatic doublets, we used a pair of commercial camera lenses (f/1.4D AF NIKKOR, Nikon, L_5, and L_6) before the camera. The actual transmission efficiency of these lenses is between T1.6 and T1.7, or a transmission efficiency between 68% to 77%. We acknowledge that these lenses might be responsible for a substantial decrease in photon collection efficiency that could be avoided by selecting alternate achromatic lenses in future designs. The reduction in photon collection efficiency caused by reflection on the galvo mirror (GVS211, Thorlab) is small, since the reflectance of each galvo mirror is at or above 99% for wavelengths ranging from 400 nm to 700 nm.

The mechanical displacement of galvomirrors introduces additional shot noise in streak imaging, because emission photons are distributed across a larger number of pixels, in comparison to widefield imaging. At the end of each streak, the galvo mirror needs to return to the starting

position for the next streak causing additional photon loss. This issue can be addressed by implementing bidirectional scanning.

2.3 Reduction in FOV

Introducing DMD reduced the FOV from 2560 $\times$ 2160 pixels^2 to 2232 $\times$ 1270 pixels^2. When driving the galvo mirrors with voltage equal to 0.04 V, the FOV size is around 91% of the original size of FOV. Under galvo voltage equal to 0.08 V, FOV size is around 84% of the original size of FOV.

2.4 Global shutter for streak imaging

High-speed streak camera recording is best performed using a global shutter, if available. Here, we used the Zyla 5.5 camera. The hardware user guide mentioned that "(under rolling shutter) the row at the top (or bottom) edge of the sensor would have started and ended its exposure $\sim$ 10 ms (1080 rows $\times$ 10 μs/row) after the rows at the center of the sensor (when using 560 MHz readout rate)."

Using rolling shutters is possible, but comes with additional challenges that need to be accounted for in the image analysis pipeline. The galvo mirror rotations are synchronized with the camera, and each rotation begins at the start of the exposure of the center row at time t_0. In this configuration, a streak at the center of the sensor will be recorded from time t_0 ms to $(t_0 + 25)$ ms. However, the streak at the top (or bottom) of the sensor will record differently: the upper part (10 ms length) of the streak will record an exposure time from $(t_0 + 25)$ ms to $(t_0 + 25 + 10)$ ms, and the lower part (15 ms length) of that streak will record from $(t_0 + 10)$ ms to $(t_0 + 25)$ ms. For streaks at different locations on the sensor, different sizes of the streak will lag by up to one frame. While this effect might be curated with a post processing algorithm, using rolling shutter might introduce extra noise that would not appear in the image using global shutters.

3 Optical Properties of the compressive microscopy

We first tested the compressive microscopy's optical properties using a fluorescent test slide (Fig. S1 (a), (b)). We displayed a grid of filled circles on the DMD and adjusted the objective height z so that the image received by the camera was in-focus. We then localized the the circles in the camera image by identifying local maxima, and we measured intensity fluctuations along the x-axis and y-axis. We captured 2D snapshots of the slide at different depths and measured the intensity change along the z-axis. We also changed the diameter of the circles and repeated the above processes (Fig. S1(a)). We observed asymmetry in the intensity curve for the z-axis . We measured the full width at half maximum (FWHM) of the intensity curves along the x, y and z-axis for different circle diameters (Fig. S1(b)). We observed that when circle diameters were less or equal to 21 pixels, the FWHM along the x-axis and y-axis were within or around 10 μm. The FWHM along the z-axis was much larger, more than 40 μm for different circle diameters.

Next, we tested the compressive microscopy's optical properties using fluorescent beads (Fig. S1 (c), (d)). We performed targeted illumination a single bead by displaying a circle pattern on the DMD. Similar to the fluorescence slide test, we changed the diameter of the circle, and we measured changes in intensity along three axes, and we computed the FWHM. In all cases, we observed that the FWHM was much smaller than what we observed in the fluorescence test slide,

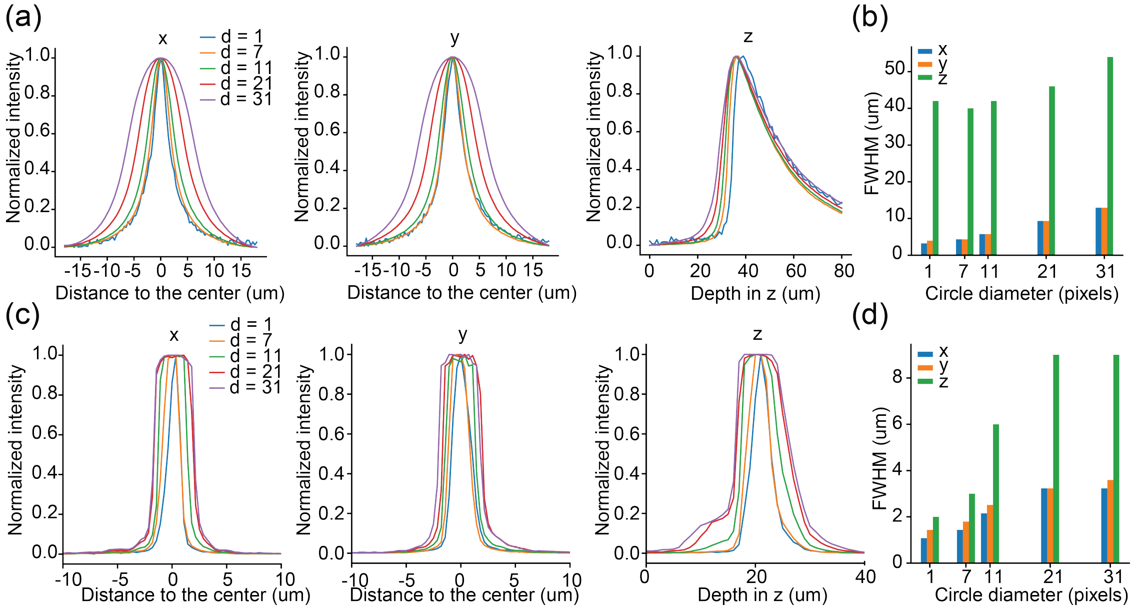

Fig S1 Optical properties of the compressive microscopy. (a)-(b) We displayed circular patterns on the DMD and illuminated the fluorescence slide. Intensity and FWHM along three axes under different circle sizes were measured. (c)-(d) We displayed a single circle pattern on DMD to perform targeted illumination on a single fluorescence bead. Intensity and FWHM along three axes under different circle sizes were measured.

because with fluorescent beads, emission fluorescence is limited to the physical dimensions of each bead.

4 Zebrafish experiment with streak imaging at 40 Hz

We controlled the galvo mirror using a sawtooth wave voltage pattern ranging from -0.04 to 0.04 V and we recorded a one-minute long streak movie at 40 Hz with a compression ratio of 10. We computed the mean image of the streak movie (Fig. S2 (a); Video 5, MP4, 7.7 MB). The recorded streak movie was more noisy in comparison to the 20Hz streak movie, since less photons were received by each pixel. We reconstructed high temporal resolution fluorescence traces at 400 Hz and compared the reconstructed traces with reference traces computed by averaging signals in each streak for each frame (Fig. S2 (b)). Finally, for neurons selected for targeted illumination (36 neurons), we computed relative fluorescence intensity on the standard deviation image of the widefield and targeted movies. Relative fluorescence intensity was equal to the maximum fluorescence of the neuron subtracted from the background fluorescence and then divided by the background fluorescence. Note the power density was different under widefield (4.2 mW·mm^{-2}) and targeted (65.4 mW·mm^{-2}) imaging. We consider an average relative fluorescence intensity above or close to 1 in the widefield movies is necessary for high quality reconstruction.

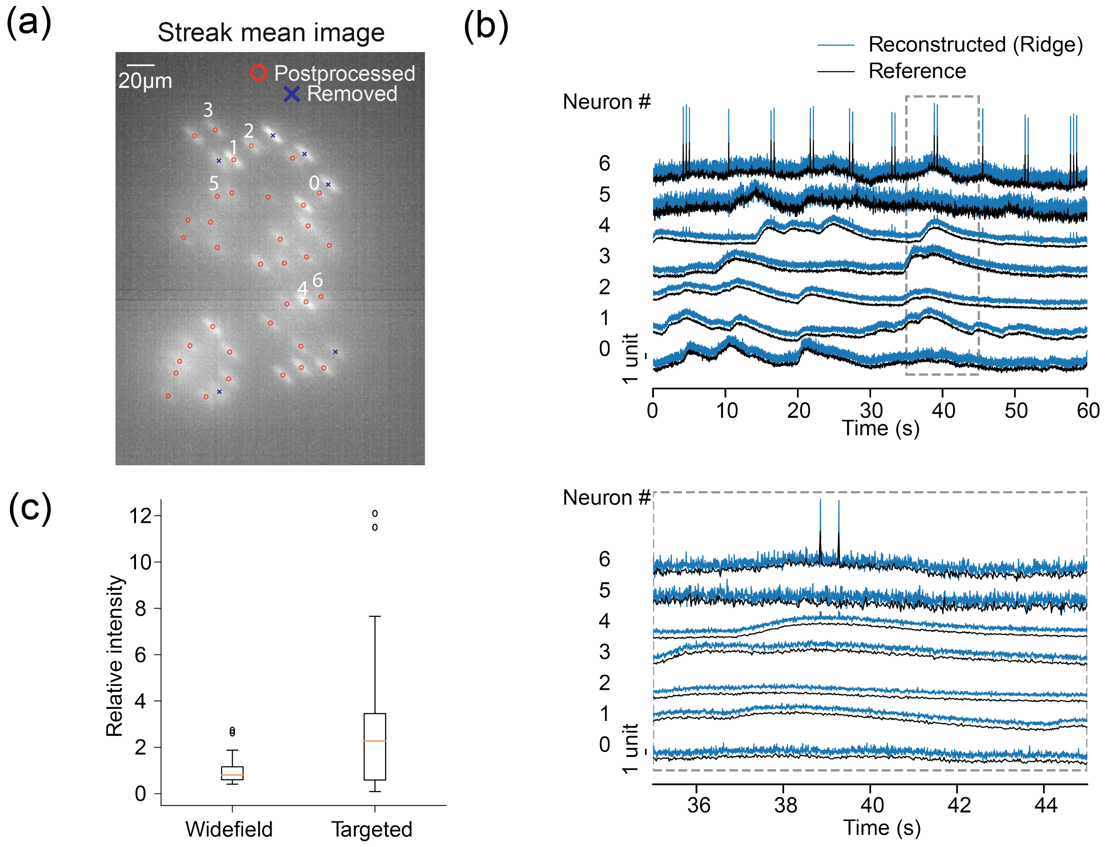

Fig S2 Recording fluorescence activity in zebrafish at 400 Hz. (a) The mean image of the streak movie is shown (Video 5, MP4, 7.7 MB). Neurons that were reconstructed for high temporal resolution signals were labeled with red circles. Neurons that suffered from optical crosstalk in targeted illumination movies were labeled with blue crosses and were not post-processed. 7 neurons were selected for showing fluorescence activity in panel (b). (b) Reconstructed fluorescence signals (blue, z-scored, 400 Hz) computed from a one-minute-long streak movie (40 Hz) across 7 selected neurons. Reference traces (black, z-scored, 40 Hz) were computed by averaging signals in each streak for each frame. Images inside dashed rectangles were magnified on the bottom. (c) For neurons selected for targeted illumination (36 neurons), we computed relative fluorescence intensity on the standard deviation image of the widefield and targeted movies. Note the LED power density was different under widefield (4.2 mW$\cdot$mm^{-2}) and targeted (65.4 mW$\cdot$mm^{-2}) imaging.
